# Prediction of chronic obstructive pulmonary disease based on multimodal data and deep learning

**DOI:** 10.1063/5.0289414

**Published:** 2025-12-24

**Authors:** Haoran Deng, Xuchun Ding, Shiping Zhu, Xue Song, Yufei Guo

**Affiliations:** Department of Respiratory and Critical Care Medicine, Hangzhou TCM Hospital Affiliated to Zhejiang Chinese Medical University, Hangzhou 310007, Zhejiang, China

## Abstract

To address the issues of insufficient utilization of multimodal information, modal heterogeneity, and data gaps leading to poor model generalization in early prediction of chronic obstructive pulmonary disease (COPD), a deep learning-based multimodal dynamic fusion network (MMDF-Net) is proposed. This model integrates chest CT images, pulmonary function indicators, and environmental exposure data. It aligns image and non-image features via a dual-tower cross-modal contrastive learning module, mitigating semantic differences across modalities. A conditional generative adversarial network is used to generate high-fidelity environmental exposure data, reducing reliance on data completeness. A dynamic gating fusion mechanism adaptively adjusts multimodal weights based on patient smoking history, age, and other attributes to suppress noise. On the COPD Gene dataset, MMDF-Net achieves an area under the curve (AUC) of 0.92, a sensitivity of 92.3%, and a specificity of 88.7%, significantly outperforming single-modal models and dynamically adjusting weights according to disease stage. These results demonstrate that this multimodal dynamic fusion strategy can effectively address data heterogeneity and individual differences, providing technical support for precise early intervention in COPD.

## I. INTRODUCTION

Chronic obstructive pulmonary disease (COPD), as a progressive respiratory disease, is a significant challenge in the field of global public health. Its pathological characteristics are persistent airflow limitation and airway inflammation. The early symptoms are hidden and easily ignored, resulting in most patients entering the middle and late stages when diagnosed, missing the best time for intervention. Current clinical screening primarily relies on pulmonary function tests and imaging. However, single-modal diagnostic methods have significant limitations. Although pulmonary function indicators can reflect the degree of airflow limitation, they are not sensitive enough to detect early structural lesions. Although imaging omics technology can capture lung morphological abnormalities, it is difficult to quantify the dynamic association between external risk factors, such as environmental exposure and genetic susceptibility, and disease progression.

The subtle early symptoms of COPD lead to late diagnosis, single-modality diagnosis has limitations, and the heterogeneity of patient clinical data further restricts the effectiveness of traditional models. This heterogeneity manifests in three aspects: significant semantic differences in the formats of computed tomography (CT) images and pulmonary function data across modalities; individual differences in disease presentation due to age and genetics; and inconsistent, and often missing, equipment and annotation standards that affect data quality. Single-modal deep learning models struggle to integrate heterogeneous features, show poor adaptability to individual differences, are sensitive to data quality, and exhibit insufficient generalization and predictive accuracy. Therefore, building an efficient multimodal fusion framework to overcome data barriers is key to achieving accurate risk stratification of COPD.^1,2^

In this paper, we present the multimodal dynamic fusion network (MMDF-Net), which integrates deep learning and multimodal data to address key issues in COPD prediction systematically and follows a three-stage approach. The first stage is the design of a cross-modal contrast alignment module with a dual-tower architecture. With Contrastive learning, we can model semantic consistency between images and features to address the conflict caused by modal heterogeneity. The second stage uses conditional generative adversarial network (C-GAN) to generate high-fidelity environmental exposure data under the constraints of clinical indicators, addressing the limitation of traditional methods' heavy reliance on data completeness. The final stage is the construction of a personalized, dynamic, gated fusion mechanism that can adaptively adjust multimodal weights based on the patient's smoking history, age, and other attributes, overcoming the limitations of static fusion strategies in terms of individual adaptability.

## II. RELATED WORK

The role of deep learning in COPD prediction primarily lies in its ability to extract complex patterns and features from large volumes of patient data, enabling doctors to make early diagnoses and deliver personalized treatment. By training deep neural network models, the system can analyze data such as lung function tests, imaging data, clinical manifestations, and predict the disease's development trend and the risk of acute exacerbation, thereby improving diagnostic accuracy and treatment outcomes. Among them, Andrea used Kaplan–Meier analysis and multivariate Cox regression to evaluate the relationship between progression of the COPD Gene score and mortality. The results showed that deep learning scores were moderately consistent with visual scores.^3^ Wu analyzed the diagnostic performance of artificial intelligence models on CT images of COPD patients, aiming to promote future research in this area. Deep learning models achieved high accuracy in diagnosing COPD.^4^ Almeida used deep learning to quantify COPD-related regional manifestations as abnormalities in a regular lung model on chest CT and to evaluate its predictive performance for disease severity. The results showed that deep learning could predict lung dysfunction and morphological deterioration.^5^ Zhuang integrated COPD-related protein–protein interaction, proteomics, and transcriptomics data through the Augmented High-Dimensional Graphical Lasso Method algorithm and developed a COPD classification prediction model.^6^ However, deep learning models have some limitations when applied to COPD prediction. These models rely heavily on data quality and quantity, and a lack of adequately labeled data or incorrect labeling may degrade model performance. Deep learning models have limited generalization and may not adapt well to data variations across regions or populations, affecting their predictive accuracy in diverse environments.

By combining different types of data for comprehensive analysis, multimodal data fusion can yield more detailed and informative results. By fusing multimodal data, missing data from specific sources can be supplemented by other sources, thereby improving the model's stability and prediction accuracy. This means that even if a particular type of data is missing, different data sources can provide sufficient information to help the model make predictions and improve its generalization.^7–9^ Robertson's study found that, compared with using each imaging method alone, a multimodal approach combining CT images, demographic data, and spirometry has been shown to improve machine-learning prediction of COPD progression.^10^ Ren developed and tested CheXMed's multimodal model, combining clinical records with image data to enhance pneumonia detection in the elderly.^11^ Multimodal data also have specific challenges. Data from different modalities often have various formats, scales, and features, requiring appropriate preprocessing and feature engineering, which increases the complexity of model training. In addition, the quality and quantity of data from different modalities may vary, which affects the effect of data fusion.

## III. EXPERIMENTAL DESIGN AND VALIDATION

**FIG. 1.**
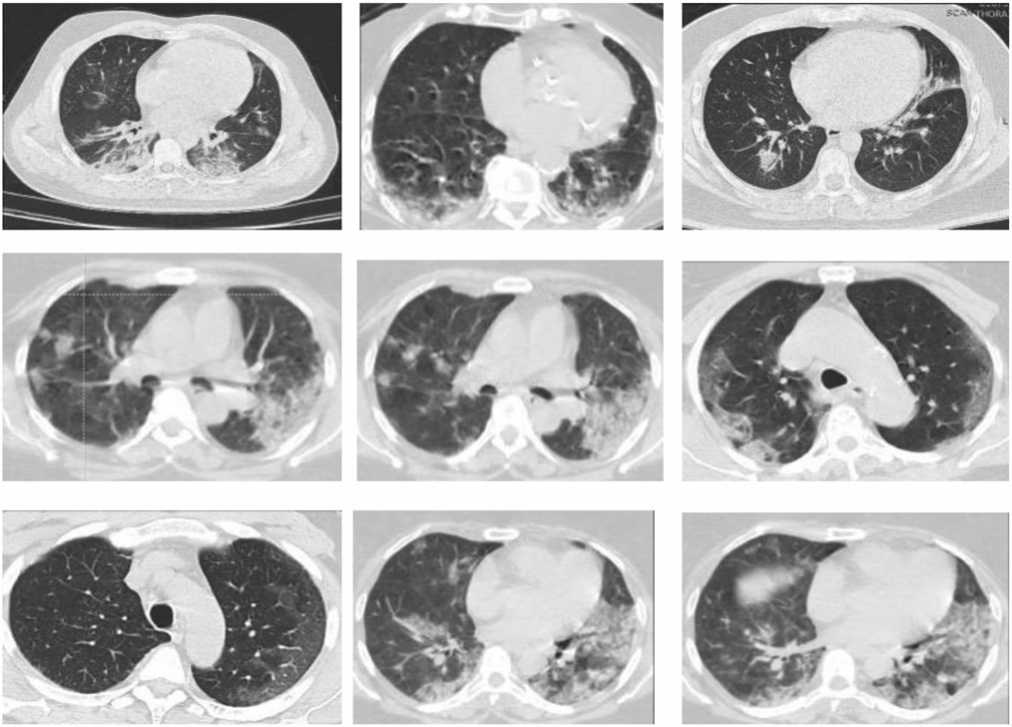
CT data images of some patients.

In this paper, we use the COPD Gene dataset, a collaboration of the National Heart, Lung, and Blood Institute and the American Lung Association, to uncover the genetic basis and pathogenesis of COPD through large-scale genomic studies. The study began in 2007 and has combined detailed clinical and genomic data from over 10 000 COPD patients and healthy controls from around the United States. The COPD Gene dataset is a valuable resource for precision medicine in the studies of COPD and will advance basic research and ultimately clinical applications of precision medicine for this disease. The dataset combines clinical data, lung function test results, genetic data, and environmental exposure data of thousands of COPD patients and healthy controls from multiple countries. CT images of some patients are shown in Fig. 1.

To simplify the calculation process, this paper selects a subset of samples for analysis. The sample selection follows the representative principle to ensure coverage of disease heterogeneity and real population characteristics. The COPD patient group is stratified by disease severity and covers key risk subgroups such as smoking, high PM2.5 exposure, and genetic susceptibility. The healthy control group is matched by age and gender to reduce confounding bias, and the data sources cover multiple regions and races to avoid single-center selection bias. Finally, 358 COPD patients and 200 healthy controls are included in the COPD Gene dataset. The relevant information is shown in Table I.

**TABLE I.** Sample characteristics information.

Characteristics	COPD patient group (n = 358)	Healthy control group (n = 200)	Statistical test (p-value)
Gender (male/female)	214/144	112/88	0.42
Age (years)	65.3 ± 8.7	63.1 ± 7.9	0.12
Forced expiratory volume/Forced vital capacity (FEV1/FVC) (%)	58.4 ± 6.2	75.6 ± 4.8	<0.001
Smoking history	282 (78.8%) (years: 35.2 ± 18.5)	45 (22.5%) (years: 4.5 ± 2.1)	<0.001
PM2.5 exposure (*μ*g/m^3^)	42.5 ± 11.3 (exposure years: 15.4 ± 5.2)	28.6 ± 8.7 (exposure years: 10.1 ± 4.8)	<0.001
Gene (SERPINA1)	89 (24.9%)	12 (6.0%)	<0.001

## IV. RESULTS

### A. Effect of cross-modal feature alignment

**FIG. 2.**
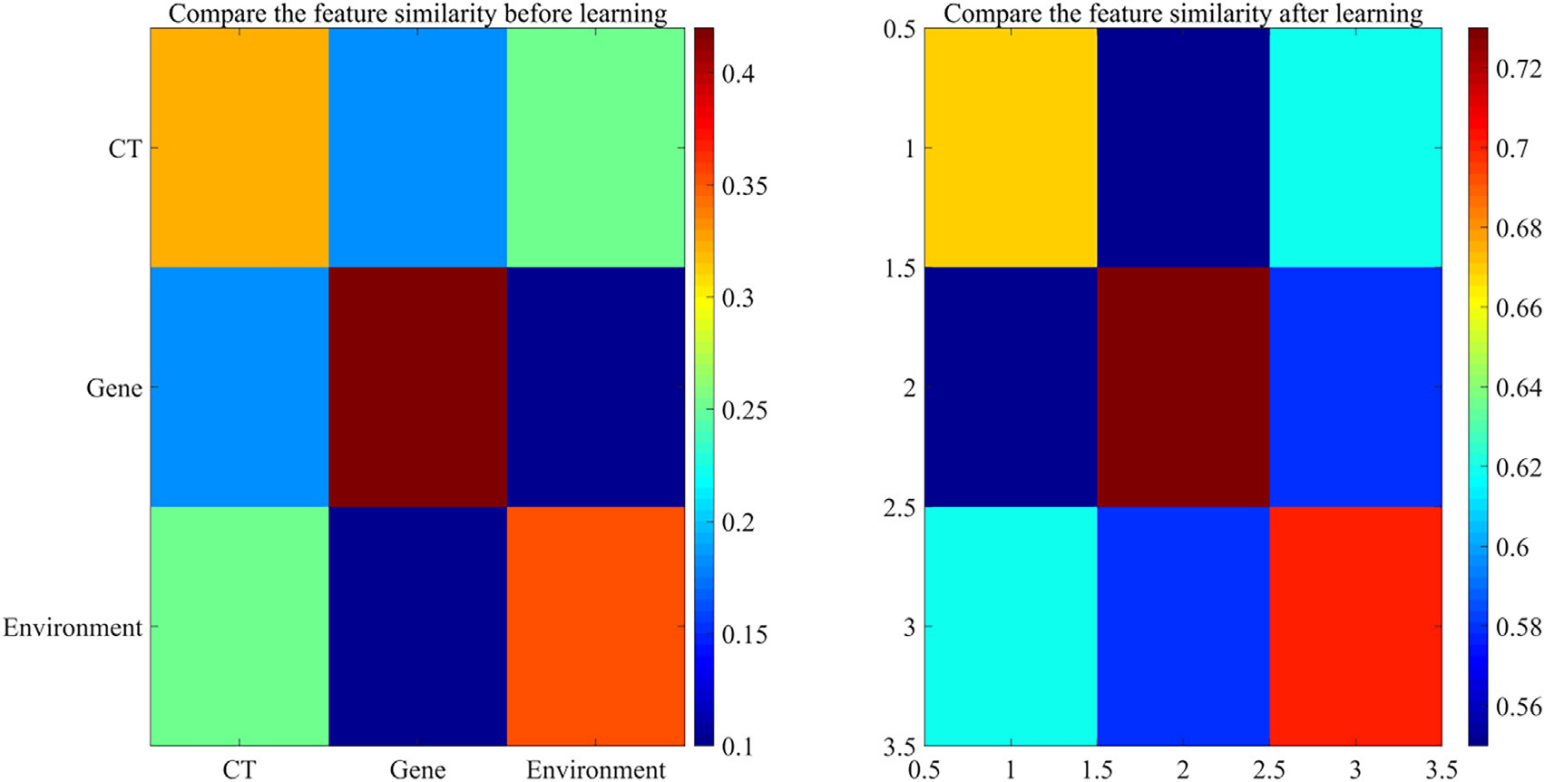
Comparison of feature similarity before and after contrast learning.

The effectiveness of the multimodal alignment module is verified by comparing the cross-modal feature similarity matrix before and after learning. The results are shown in Fig. 2:

The results in Fig. 2 show that after contrast learning, the cosine similarity between CT image features and gene sequence features increases from 0.32 to 0.67 (p < 0.001), and the distribution difference between modalities decreases from 0.85 to 0.21, indicating that cross-modal contrast learning significantly enhances feature semantic consistency.

**FIG. 3.**
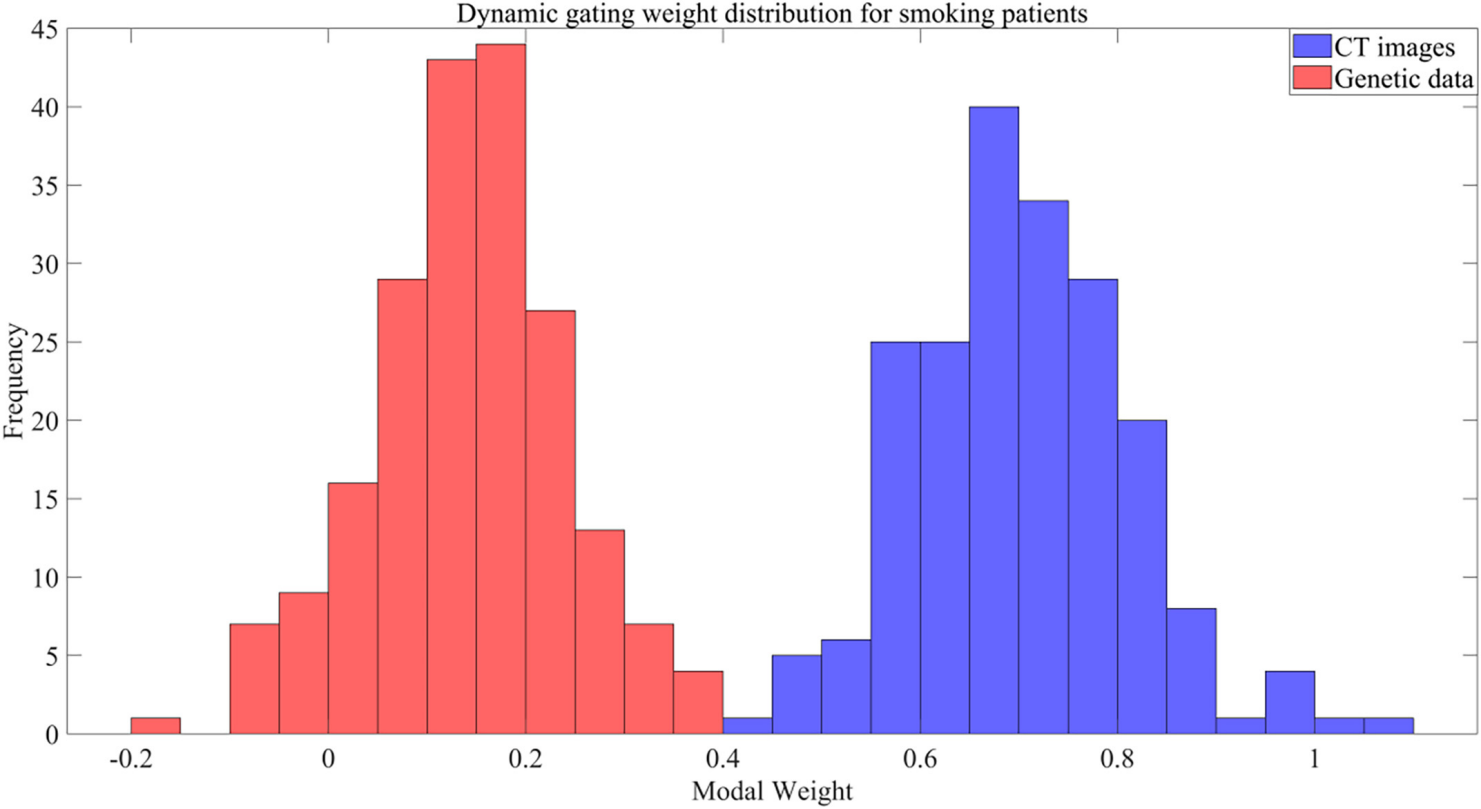
Weight distribution of different modalities.

By analyzing the weights of modal data, it is found that there is significant heterogeneity in the weight distribution of different modalities, as shown in Fig. 3:

As shown in Fig. 3, the median CT weight for smokers is 0.72, whereas the gene weight is only 0.15, indicating heterogeneity in the weight distributions across modalities. MMDF-Net achieves efficient multimodal data fusion via cross-modal contrast learning and a dynamic weight control mechanism. Feature alignment in contrastive learning addresses the semantic gap caused by modal heterogeneity, while weight allocation improves model robustness by suppressing noise modalities.

To verify the role of different modules in the system, an ablation experiment is conducted by gradually removing them to evaluate performance. The results are shown in Table II.

**TABLE II.** Comparison of module ablation experiments.

	AUC	Sensitivity (%)	Specificity (%)	F1	Precision	Recall
Without a contrastive learning module	0.86	84.2	79.1	0.83	0.82	0.84
Without the generation module	0.88	86.1	81.5	0.85	0.85	0.86
Without a dynamic gating mechanism	0.86	87.1	82.6	0.86	0.86	0.87
Complete model	0.92	92.3	88.7	0.91	0.91	0.92

According to Table II, MMDF-Net achieves the highest performance across the whole configuration of contrastive learning, C-GAN, and a dynamic gating mechanism, with an AUC of 0.92 and an F1 score of 0.91, surpassing all models with the modules removed. The model without the contrastive learning mechanism achieved an AUC of 0.86, demonstrating the importance of this mechanism for cross-modal semantic alignment. Removing the C-GAN yielded an AUC score of 0.88, indicating that the generative component contributes positively to completing missing or incomplete information in environmental data. Finally, eliminating dynamic gating reduces the model's flexibility and weakens performance. Overall, these three modules synergize to enhance the model's robustness, accuracy, and generalization, confirming the reasonableness and practicality of the MMDF-Net's structural design for multimodal COPD prediction.

### B. Performance differences of different modalities in prediction

To verify the necessity of multimodal data fusion, this paper evaluates the performance differences of single-modal (CT images, lung function indicators, and environmental exposure data) and multimodal fusion models (MMDF-Net) in COPD prediction. The results are shown in Table III.

**TABLE III.** Prediction performance of different modalities in COPD.

Modal type	AUC	Sensitivity (%)	Specificity (%)	Statistical tests	95% Confidence interval
CT images	0.85 ± 0.03	82.1 ± 4.2	80.3 ± 3.8	t = 18.7, p < 0.001	[0.81, 0.89]
FEV1/FVC	0.78 ± 0.05	75.6 ± 5.1	76.8 ± 4.5	t = 12.3, p < 0.001	[0.73, 0.83]
Environmental exposure data	0.65 ± 0.08	62.0 ± 7.3	58.0 ± 6.9	t = 6.9, p > 0.001	[0.59, 0.71]
MMDF-Net	0.92 ± 0.02	92.3 ± 2.8	88.7 ± 3.1	t = 24.1, p < 0.001	[0.89, 0.95]

Table III shows the performance differences across modalities for COPD prediction. MMDF-Net achieves the highest AUC of 0.92, with sensitivities and specificities of 92.3% and 88.7%, respectively, which are significantly better than those of other methods. CT images perform second, with an AUC of 0.85, and sensitivity and specificity of more than 80%, showing strong diagnostic ability. FEV1/FVC, as a traditional lung function indicator, has an AUC of 0.78. Although its performance is slightly inferior to CT images, it still has specific clinical reference value. In contrast, environmental exposure data have the weakest prediction effect, with an AUC of only 0.65, and relatively low sensitivity and specificity. The statistical test results show that, except for the environmental exposure data, the p-values for the remaining modalities are all less than 0.001, indicating that their predictive performance is statistically significant.

The outstanding performance of MMDF-Net may be due to its multimodal fusion design, which effectively leverages information from multiple data sources to improve prediction accuracy. CT images have high practical value in diagnosing COPD because they directly reflect changes in lung structure. Still, they have the disadvantage of being limited in their ability to fully capture functional changes or the influence of the external environment. FEV1/FVC is a classic lung function evaluation indicator. Although it is widely used, it can only reflect airflow limitation and cannot fully capture the complex characteristics of the disease, so its performance is slightly limited. The inefficient prediction of environmental exposure data may be related to its indirectness and non-specificity. The impact of ecological factors on COPD often requires long-term exposure, and there are substantial differences between individuals. It is evident that multimodal fusion technology, such as MMDF-Net, has excellent potential in COPD prediction. Careful consideration of imaging, functional indicators, and evaluation methods for environmental factors can help more comprehensively understand the pathogenesis of the disease and its risk factors.

To further analyze the predictive ability of each modality across different disease stages, Table IV compares the feature importance of the modalities in patients with mild, moderate, and severe COPD.

**TABLE IV.** Modal weight distribution of patients at different stages.

Disease stage	CT image weight	Lung function weight	Environmental exposure weight
Mild COPD	0.71 ± 0.12	0.23 ± 0.08	0.06 ± 0.03
Moderate COPD	0.69 ± 0.11	0.25 ± 0.09	0.06 ± 0.04
Severe COPD	0.22 ± 0.15	0.68 ± 0.10	0.10 ± 0.05

The results show that CT images have a higher weight (0.71) in patients with mild and moderate COPD. In contrast, the weight of lung function indicators increases to 0.68 in patients with severe COPD, suggesting greater sensitivity of these indicators after a compensatory decline in lung function. The data in Table IV show that the model needs to adjust the modal weight as the disease progresses dynamically. Mild and moderate patients rely on imaging features to capture early structural abnormalities. In contrast, severe patients have significant deterioration of lung function, and lung function indicators become the primary basis for discrimination. Environmental exposure data are slightly higher in severe patients because they reflect long-term risks.

### C. Risk specificity of different modalities

To explore the specific role of multimodal features in risk stratification, this paper further analyzes the differences in modal weights in smoker and nonsmoker subgroups.

**FIG. 4.**
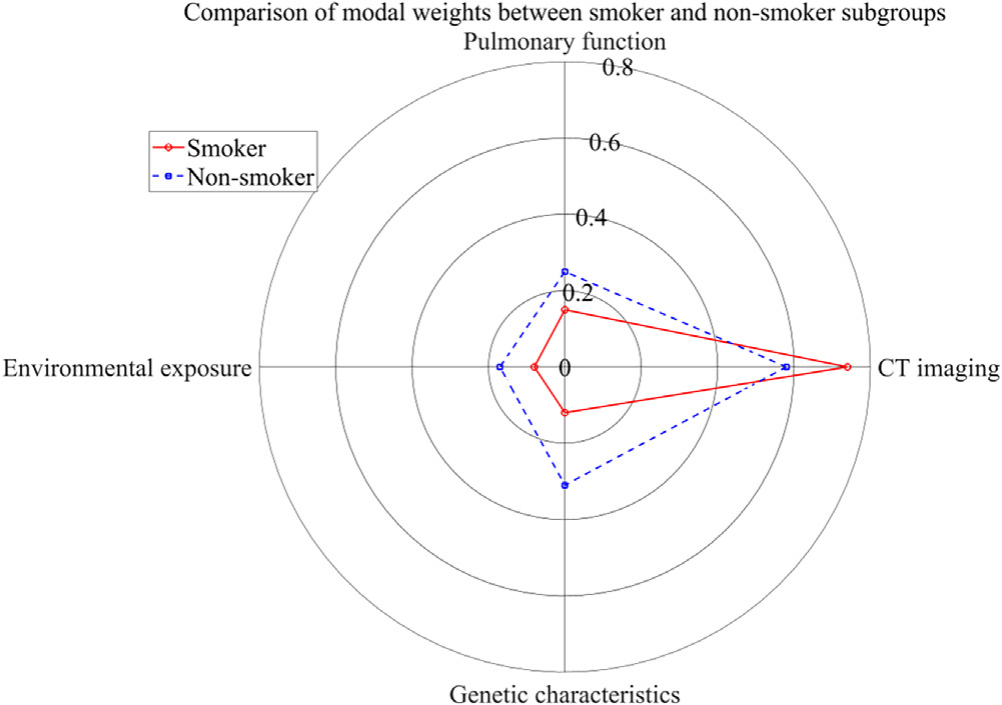
Modal weights in smoker and nonsmoker subgroups.

Figure 4 shows the differences in modal weights across subgroups. Smokers exhibit significant lung structural damage due to long-term tobacco exposure, and CT images become the main discriminant feature; genetic or environmental factors, and the importance of genetic and ecological data increases may be a more important driver of COPD. The reason for this difference is that smoking directly causes lung morphological changes, and CT can intuitively capture such abnormalities; the etiology of nonsmokers is complex and requires a comprehensive evaluation based on genetic and environmental data. The MMDF-Net model adjusts modal weights based on smoking history, improving its adaptability across subgroups and providing personalized screening recommendations for clinicians: smokers are prioritized for CT examinations, while nonsmokers are prioritized for genetic testing or environmental exposure evaluations.

In addition, the modal weight comparison of PM2.5 exposure groups is analyzed, and the results are shown in Table V.

**TABLE V.** Modal weights of PM2.5 exposure groups.

PM2.5 exposure group	CT image weight	Lung function weight	Environmental exposure weight
High exposure (≥40 *μ*g/m^3^)	0.63 ± 0.10	0.19 ± 0.07	0.18 ± 0.06
Low exposure (<40 *μ*g/m^3^)	0.70 ± 0.12	0.25 ± 0.08	0.05 ± 0.03

As shown in Table V, the analysis of stratified PM2.5 exposure indicates that the weight of environmental data is more pronounced in the high-exposure group than in the low-exposure group. High exposure to PM2.5 exacerbates lung injury through a long-term inflammatory response. Ecological data and CT images together serve as the main features for risk prediction. At the same time, the low-exposure group is insulated from the environment and therefore relies more on CT or lung function measurements.

### D. Performance comparison of different algorithms

To verify the superiority of MMDF-Net, MMDF-Net is compared with mainstream algorithms, including single-modal models and multimodal fusion methods. The results are shown in Table VI.

**TABLE VI.** Performance comparison of different algorithms.

	AUC	Sensitivity (%)	Specificity (%)	F1	Precision	Recall
ResNet-50	0.85	82.1	80.3	0.81	0.80	0.82
Support vector machine (SVM)^36^	0.78	75.6	76.8	0.75	0.75	0.75
Max-relevance and min-redundancy (mRMR)^37^	0.87	83.4	81.0	0.82	0.82	0.83
Uncertainty-guided graph attention network (UG-GAT)^38^	0.89	86.7	83.2	0.85	0.85	0.87
CheXMed^11^	0.88	85.3	82.5	0.84	0.84	0.85
MMDF-Net	0.92	92.3	88.7	0.91	0.91	0.92

As shown in Table VI, MMDF-Net outperforms existing models across all evaluation metrics and demonstrates excellent predictive performance. Specifically, MMDF-Net's AUC is 0.92; F1-score is 0.91; and Precision and Recall are 0.91 and 0.92, respectively, which are significantly better than those of the comparison model. Compared with the suboptimal algorithm UG-GAT, MMDF-Net improves AUC by three percentage points, and sensitivity and specificity are increased by about 5%, respectively. Traditional single-modal methods, such as ResNet-50 and SVM, perform poorly across all indicators, underscoring the limitations of a single data source for the complex characterization of COPD. Although fusion strategies such as CheXMed and mRMR improve performance to a certain extent, they lack dynamic adaptability and mechanisms for handling missing data, resulting in limited generalization and stability. In summary, MMDF-Net effectively improves feature fusion quality and prediction reliability through dynamic gating and multimodal alignment mechanisms and is suitable for integrated analysis of multi-source heterogeneous medical data.

## V. DISCUSSION

The MMDF-Net developed in this study offers a technological solution for predicting COPD. By incorporating chest CT images, pulmonary function metrics, and environmental exposures, the model demonstrates considerable improvements in predictive precision through its innovative cross-modal feature alignment and dynamic weight distributions. While traditional single-modal models lack precision, MMDF-Net's primary advantage is its greater adaptability compared to other static methods. Due to differences in etiology (smokers vs nonsmokers), MMDF-Net's gating mechanism prioritizes CT images and genomic data over environmental exposure data. Prioritizing these modalities addresses the heterogeneity issue in our multimodal data while also providing a theoretical basis for developing personalized screening approaches.

This study also has certain shortcomings. First, the experimental data are entirely based on the COPD Gene dataset. Although it covers multiple regions and races, the lack of independent external verification may weaken the model's generalization. In actual clinical environments, differences in data-acquisition equipment, inconsistent annotation standards, and noise interference (e.g., respiratory motion artifacts) may affect model performance. Second, the architectural complexity of MMDF-Net requires high computing resources, which may limit its application in primary medical scenarios. In the future, computing efficiency can be optimized through lightweight technology, or hardware thresholds can be lowered by using a distributed computing framework.

From a practical perspective, MMDF-Net's high sensitivity and dynamic weight adjustment capabilities are highly valuable. In mild and moderate patients, the model identifies early lung structural abnormalities through high-weight CT features, which helps promote preventive intervention; in severe patients, the significant increase in the weight of lung function indicators can assist in evaluating disease progression and treatment response. In addition, the model's increased weight on environmental data for people with high PM2.5 exposure not only verifies the key role of environmental factors in COPD onset but also provides data-driven decision support for public health policies.

To address real-world scenarios in clinical applications, such as data gaps and equipment differences, a layered adaptation approach can enhance MMDF-Net's robustness. First, regarding data gap adaptation, if environmental exposure data are mildly missing, multiple imputation can be introduced during the C-GAN generation stage to initialize noise vectors and optimize the generated distribution by combining real data fragments. If lung function indicators are severely missing, modality missing markers can be added to the dynamic gating mechanism to automatically increase the weight ratio of CT images and genetic data, and modality dropout training can enhance the model's tolerance to partial modality missingness. Second, regarding equipment differences, to address resolution differences across CT models, a cross-device histogram-matching step can be added during image preprocessing to unify the grayscale distribution range. For image noise issues with low-configuration equipment in primary healthcare institutions, a lightweight noise reduction module can be embedded before the ResNet-50 encoder, and transfer learning can be used to fine-tune the pre-trained weights from high-configuration equipment to the low-configuration data domain, meeting the needs of multi-scenario clinical deployment.

Future research can be deepened from three aspects: First, the dimension of multimodal data is expanded, and metabolomics or epigenetic data are included to analyze the metabolic abnormalities and epigenetic regulation mechanisms of COPD; second, the cross-disease prediction framework is explored, and the migration potential of MMDF-Net in respiratory diseases such as asthma or pulmonary fibrosis is verified; third, a multi-center collaborative platform is constructed to improve the model's robustness and clinical universality by integrating heterogeneous clinical data.

## VI. CONCLUSIONS

CT images and clinical information are essential bases for diagnosing COPD. Quantitative analysis of these data can achieve early diagnosis of COPD. However, the subtle differences in lung diseases in imaging, the texture characteristics of the complex structure of lung tissue morphology at limited resolution, and the unstructured, diverse, and ambiguous characteristics of clinical diagnostic information, coupled with the problem of synchronous association between clinical data, have brought significant challenges and difficulties to the precise quantitative analysis of COPD. In this paper, a multimodal fusion framework is proposed to address the problems of modal heterogeneity and missing data in COPD prediction. By combining chest CT images, lung function indicators, and environmental exposure data, contrastive learning is used to eliminate feature differences across modalities, and missing information is filled in using conditional generative adversarial networks. The model applies a dynamic weight allocation mechanism that adaptively adjusts the feature fusion ratio based on the patient's smoking history, age, and other attributes, achieving high-precision predictions with an AUC of 0.92 and a sensitivity of 92.3% on the data, and showing significant advantages, particularly among mild-to-moderate patients.

## VII. METHODS

### A. Dual-tower architecture feature alignment

**FIG. 5.**
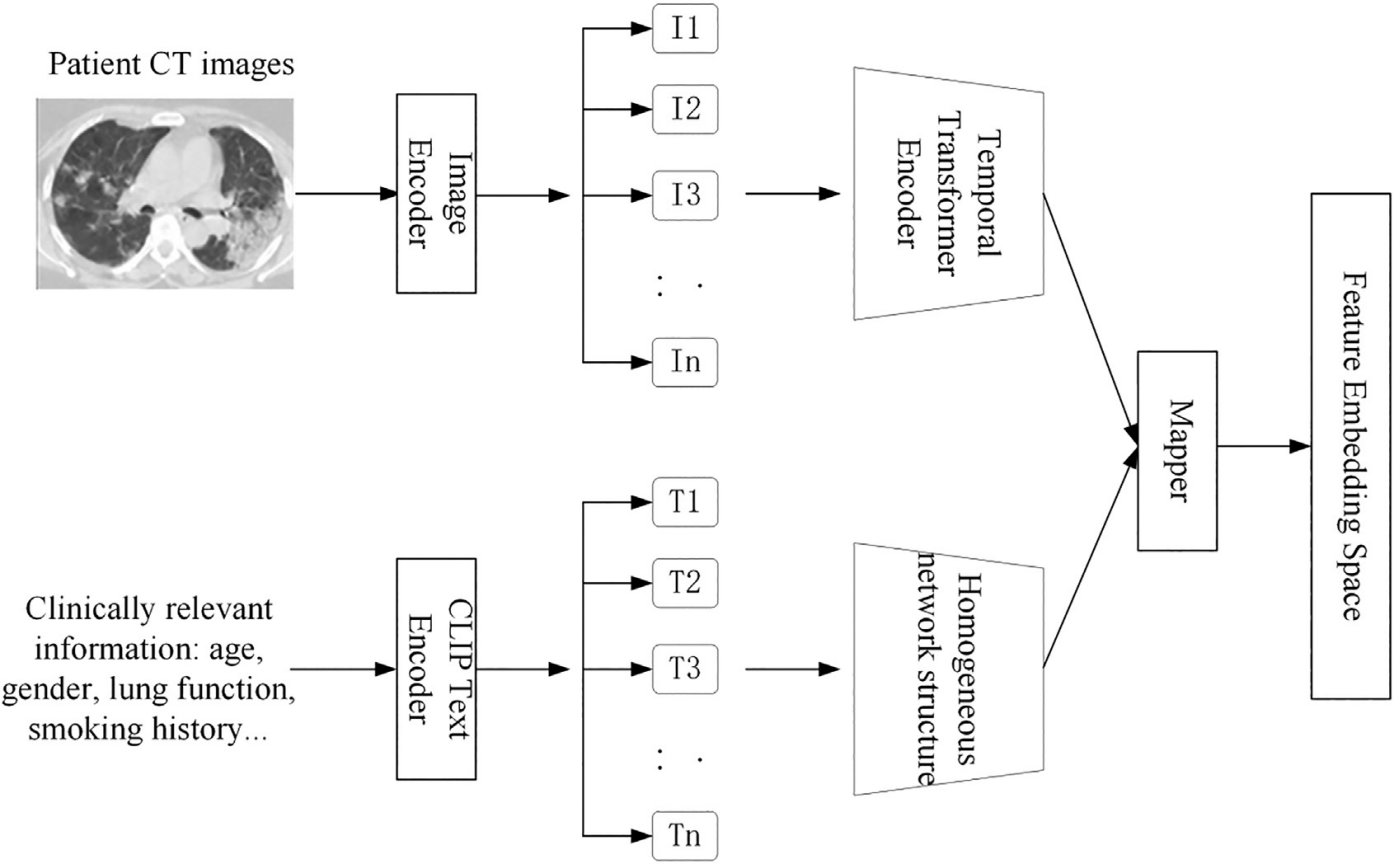
Data alignment process.

The traditional way of combining text and images to train artificial intelligence (AI) is to provide feedback to an AI model by assigning images to predefined categories.^12^ With conventional training practices, image encoders can only associate or combine features within an image with predetermined categories. As a result, while explicitly supervised training can increase an AI model's ability to identify pictures in closed domains, it also restricts the model's semantic space, thereby limiting its ability to generalize to open-domain concepts. The contrastive language-image pretraining (CLIP) model uses two parallel encoders to process visual and verbal data. Therefore, it is possible to achieve better performance than the standard approach with a single network. The CLIP architecture uses a natural language visual content supervision framework. Its basic structure consists of two independent streams of encoding networks,^13^ as shown in Fig. 5. The visual branch encodes the input image into a feature vector using ResNet-50, and the text branch processes any natural-language description using a homogeneous network structure.^14,15^ During the training process, a similarity matrix of image-text pairs is constructed, and a contrastive learning strategy is implemented in the feature space, enabling the visual representation and semantic expression to adapt dynamically and overcoming the limitations of the traditional fixed-label system.

Imagine each training batch contains N pairs of images and text. After the image encoder and text encoder, there will be N image embedding representations and N text embedding representations. The embedding representations will be mapped to a common embedding space via an embedding projection matrix, enabling alignment and similarity calculation between image and text embeddings. After they are projected into the common embedding space, all embedding vectors will be L2-normalized, which stabilizes and improves the effectiveness of the similarity calculations. That is, every embedding vector will have length 1, i.e., each embedding vector in the common space will be standardized to length 1, as follows:

$$I_{e}^{\text{norm}}=\frac{I_{f}*W_{i}}{{\left\|I_{f}*W_{i}\right\|}_{2}},$$ (1)

$$T_{e}^{\text{norm}}=\frac{T_{f}*W_{t}}{{\left\|T_{f}*W_{t}\right\|}_{2}}.$$ (2)

Here, $I_{f}$ and $T_{f}$ are the image and text feature matrices, and $W_{i}$ and $W_{t}$ are the projection matrices of the image and text. By calculating the cosine similarity between the image embedding and the text embedding, an N × N similarity matrix is generated. The rows of the similarity matrix represent the embedding representation of the image, and each row corresponds to the embedding representation of an image. The columns of the matrix represent the embedding representation of the text description, and each column corresponds to the embedding representation of a text. Each element of the matrix reflects the semantic similarity between the corresponding image and text description. The cosine similarity is computed as follows:

$$c\left(I_{i},T_{j}\right)=\frac{\sum_{k=1}^{d}I_{i},T_{j},k}{\sqrt{\sum_{k=1}^{d}I_{i,k}^{2}}*\sqrt{\sum_{k=1}^{d}T_{j,k}^{2}}}.$$ (3)

Here, $I_{i}$ is the i th image embedding vector, and $T_{j}$ is the j th text embedding vector. Afterward, the model is trained using contrastive learning. The positive samples of contrastive learning are correctly paired images and text descriptions, which are located on the diagonal of the similarity matrix. Negative samples are incorrectly matched pictures and texts, which appear in the off diagonal of the similarity matrix. For each image-to-text pair, the cross-entropy loss function is used to calculate the image-to-text and text-to-image losses, respectively, and the average is taken to effectively guide the model to continuously adjust parameters during the training process and improve its ability to capture the semantic association between images and texts. The corresponding computations are performed according to the following expressions:

$${\text{loss}}_{I}=\frac{1}{N}\sum_{i=1}^{N}-\text{log}(\frac{\text{exp}(S_{ii}/T)}{\sum_{i=1}^{N}\text{exp}(S_{ii}/T)}),$$ (4)

$${\text{loss}}_{T}=\frac{1}{N}\sum_{j=1}^{N}-\text{log}\left(\frac{\text{exp}\left(\frac{S_{jj}}{T}\right)}{\sum_{j=1}^{N}\text{exp}(\frac{S_{jj}}{T})}\right),$$ (5)

$$\text{loss}=\frac{{\text{loss}}_{I}+{\text{loss}}_{T}}{2}.$$ (6)

Here, S is the similarity matrix, and T is the temperature parameter that controls the distribution of similarity values. i and j represent indices, and $\text{exp}(S_{ii}/T)$ and $\text{exp}(S_{jj}/T)$ represent the similarity values of the positive sample pairs. By maximizing the similarity between positive samples and minimizing it between negative samples, the model can effectively learn the true association between images and texts.

### B. Data processing

#### 1. Image data processing

Chest CT is a standard imaging modality for diagnosing pneumonia. CT technology is based on a set of attenuation coefficients derived from the two-dimensional distribution matrix of organ slices. This information is combined and rearranged into different pixel grayscale values to form a CT image based on clinical information and image size. The denser the CT image pixels, the greater the improvement in image details, features, and spatial resolution.^16,17^ Because human organs, such as the lungs and liver, are similar in density to muscle-related soft tissues and have an absorption rate identical to that of water, they are often used as substitutes for these tissues. CT technology can demonstrate the entire structure of organs and bones, including the lungs.^18,19^

Histogram Equalization (HE) is a method that uses histogram statistics to modify the histogram. It can effectively process the histogram of the original image, ensuring that each gray level has a uniform probability distribution. It can automatically improve the overall picture contrast by adjusting the dynamic range of the image's grayscale values, resulting in a higher contrast and allowing most details to be seen.^20,21^ This paper uses HE to enhance CT images. The classic histogram analysis method is as follows: The input histogram is described by H(p); the input grayscale range is [p0, pk], and the goal is to find a monotonic pixel brightness transformation q = T (p) so that the output histogram G(p) is uniform in the entire output brightness range [p0, pk].^22^ The histogram can be considered as a discrete probability density function such that

$$\sum_{i=0}^{k}G(q_{i})=\sum_{i=0}^{k}H(p_{i}).$$ (7)

The sum in Formula (7) can be understood as the accumulation of discrete probability density functions. Assuming that there are M rows and N columns of pixels in an image, the equalized histogram G(p) corresponds to the equalized discrete probability density function f, whose value is a constant,

$$f=\text{MN}/(q_{k}-q_{0}).$$ (8)

The value of Formula (8) is replaced with the left side of Formula (7), so that the precisely equalized histogram can be obtained. Then, Formula (7) becomes

$$\text{MN}\int_{q_{0}}^{q}\frac{1}{q_{k}-q_{0}}\text{ds}=\frac{\text{MN}(q-q_{0})}{q_{k}-q_{0}}=\int_{p_{0}}^{p}H(s)\text{ds}.$$ (9)

In this way, the image brightness transformation is obtained, and expressed as follows:

$$q=T\left(p\right)=\frac{q_{k}-q_{0}}{\text{MN}}\int_{p_{0}}^{p}H\left(s\right)\text{ds}+q_{0}.$$ (10)

The integral in Formula (10) is called the accumulated histogram, which is usually approximated by superposition, and the obtained histogram is not entirely equal. For the discrete case, the approximation of the continuous pixel brightness transformation in Formula (10) is shown in the following expression:

$$q=T(p)=\frac{q_{k}-q_{0}}{\text{MN}}\int_{p_{0}}^{p}H(s)\Delta s+q_{0}.$$ (11)

**FIG. 6.**
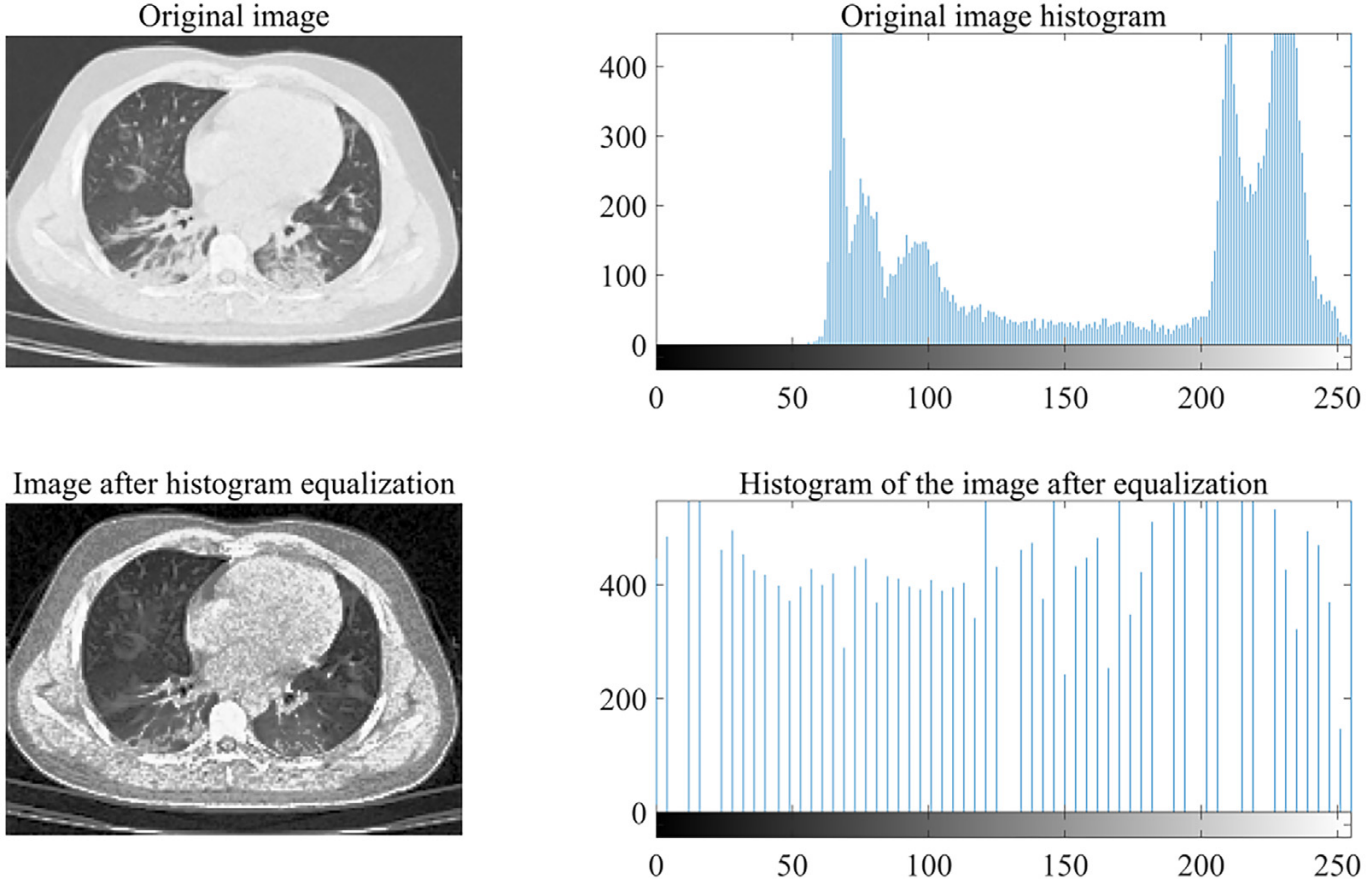
Histogram enhancement.

This paper uses the histogram equalization image enhancement algorithm for enhancement.^23^ The images before and after histogram equalization are shown in Fig. 6.

It can be seen that before histogram enhancement, the overall image is darker. However, after equalization and enhancement, the number of occurrences of each contrast is increased, and the image clarity is improved to a certain extent.

At present, there is a gradient vanishing problem in image processing, and ResNet-50 can effectively address it.^24,25^ This paper uses ResNet-50 to process the image. First, the preprocessed image is standardized. Assuming that the input image is I(x,y), with its pixel value in the range [0, 255] or other intervals, it is standardized as follows:

$$I_{\text{norm}}(x,y)=\frac{I(x,y)-\mu }{\sigma }.$$ (12)

Here, μ and σ represent the mean and standard deviation of all images in the training set, respectively. This standardization operation not only helps to accelerate model convergence but also reduces the impact of lighting or device differences.

In the CLIP dual-tower architecture model, ResNet-50 is used as the image encoder. ResNet-50 can capture rich low-level and high-level visual features.^26,27^ For the feature extraction task on CT images, the pre-trained ResNet-50 model can be loaded directly, and its last fully connected layer can be removed while retaining its feature-extraction capabilities.

The pre-trained ResNet-50 model architecture is organized into four main parts.^28,29^ The first stage uses a 7 × 7 convolution followed by a 3 × 3 maximum pooling operation to effectively reduce image size and enable a more compact data representation for the next step. The second stage deepens the exploration of high-level image features by leveraging a residual structure in the residual blocks, which are divided into Conv2, Conv3, Conv4, and Conv5.

**FIG. 7.**
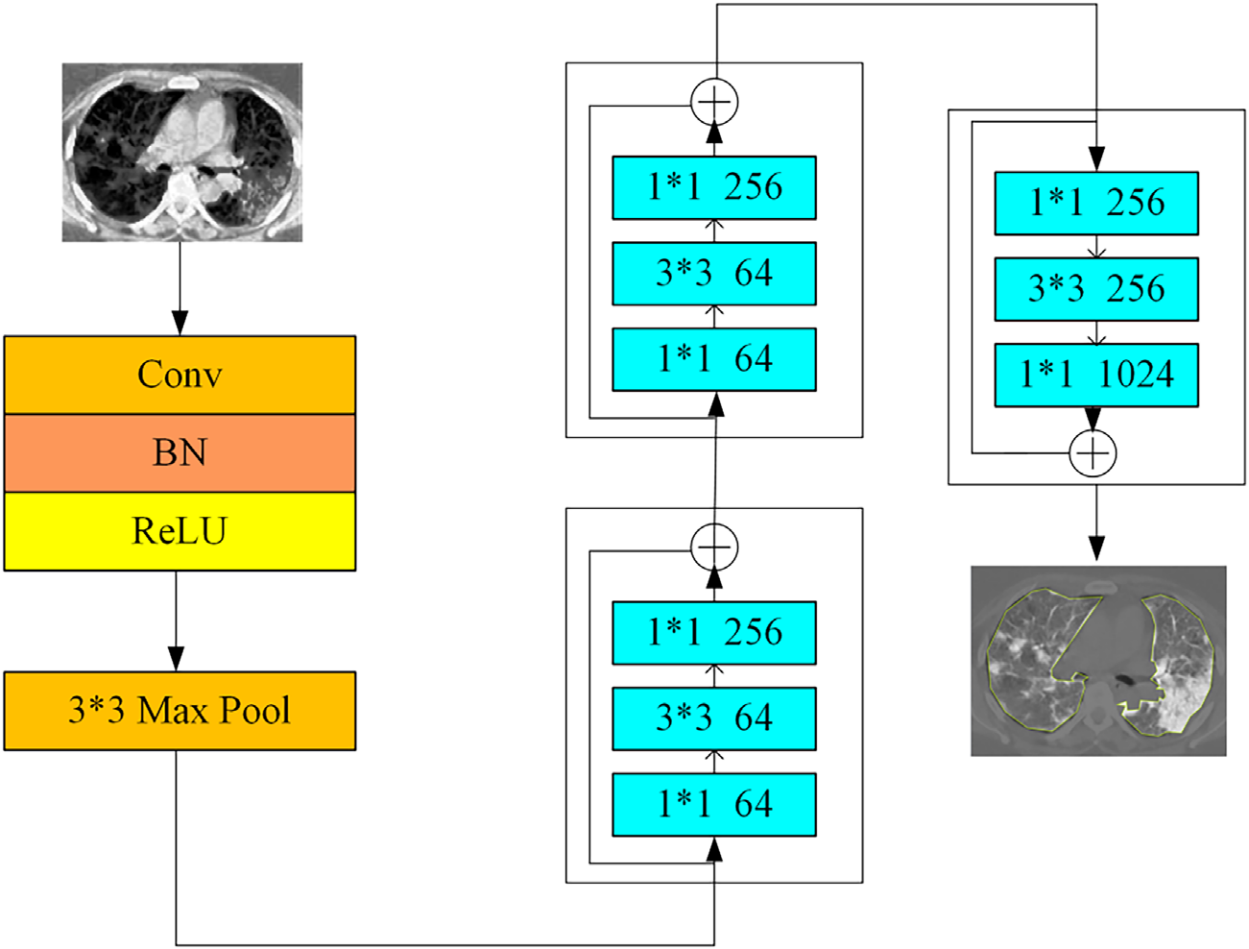
ResNet-50 architecture.

As shown in Fig. 7, the preprocessed CT images are sequentially fed into ResNet-50 for feature extraction. Assuming that the size of the input image is H*×W*C, after the multilayer convolution and pooling operations of ResNet-50, the output is a fixed-length feature vector, and each CT image can get a corresponding feature vector.

#### 2. Text data processing

The text branch processes clinical information for patients, including smoking history, lung function indicators (FEV1/FVC), acute exacerbation frequency, gender, age, and other data. This branch aims to encode clinical variables from non-image modalities into semantic embeddings of the same dimension as visual features, thereby achieving cross-modal feature alignment.

Since the original clinical data exist in the form of structured tables and does not have the contextual dependency of natural language, this paper adopts a processing method equivalent to the natural language processing task, treating the structured variables as discrete representations of “text tokens” and mapping them to a continuous vector space through a unified embedding mechanism. In this process, categorical variables (gender, smoking status) are converted into sparse vectors through one-hot encoding. In contrast, continuous variables (age, FEV1/FVC) are first Z-score standardized to ensure that each feature is within a relatively balanced numerical range, as follows:

$$x^{\prime }=\frac{x-\mu }{\sigma }.$$ (13)

Here, x is the original value, and μ and σ are the mean and standard deviation of the variable in the training set. Categorical variables are converted into discrete vectors through one-hot encoding.

In selecting an encoder, a multilayer perceptron is used to simulate the text encoder network. Its structure consists of several fully connected layers and nonlinear activation functions, and it has strong nonlinear fitting and expressive capabilities. The encoder can be regarded as an isomorphic mapping of the CLIP text branch in the structured data scenario, and it finally outputs a fixed-dimensional semantic embedding in the same embedding space as the image feature vector generated by the visual branch. The calculation is performed according to the following formula:

$$t=\text{MLP}(c)=f_{3}(W_{3}f_{2}(W_{2}f_{1}(W_{1}c+b_{1})+b_{2})+b_{3}).$$ (14)

Here, $f_{i}$ represents the activation function, and $W_{i}$ is the bias parameter.

To align with the visual features, the text embedding should be linearly transformed by a set of learnable projection matrices after passing through the primary encoder, ensuring that different modalities share a unified scale in the semantic space. Subsequently, the embedding is L2-normalized and mapped to the unit sphere to meet the normative requirements for cosine similarity-based contrastive learning. The normalization is performed according to the following formula:

$$t^{\prime }=\frac{W_{t}t}{\left\|W_{t}t\right\|}.$$ (15)

Here, $W_{t}\in R^{d*d}$ is the learnable projection matrix, and the output t is the normalized text modal embedding.

During training, the system builds batch-level image–text pairs, computes a similarity matrix of image and text embeddings, and uses the cross-entropy loss function to supervise and optimize the positive and negative samples. This mechanism effectively enhances the model's ability to capture the semantic relationship between image and non-image features, improves the robustness and consistency of semantic modality fusion, and establishes a solid foundation for subsequent multimodal feature integration.

### C. Environmental exposure data generation

To mitigate the adverse effects of missing environmental exposure data on the performance of multimodal models, a conditional generative adversarial network (C-GAN) is applied to generate high-fidelity ecological data. This method uses the patient's clinical indicators as *a priori* conditions. It captures the potential nonlinear association between individual characteristics and environmental exposure through conditional modeling, thereby achieving data missing completion and feature expansion.^30^

In terms of model structure, C-GAN consists of a generator and a discriminator. The generator takes the clinical condition vector and a concatenation of the clinical condition vector and Gaussian noise as input, uses a multilayer perceptron for feature transformation, and finally outputs the fitted environmental exposure variable. Clinical conditions include Z-score standardization of continuous variables and unique-hot encoding of categorical variables to ensure that different types of features are effectively modeled at a unified scale.

To improve training stability, the discriminator is trained to distinguish between real and generated samples.^31^ An enhanced Wasserstein GAN with Gradient Penalty (WGAN-GP) is adopted, which alleviates the problems of mode collapse and gradient instability in traditional GAN training. The corresponding computation is performed according to the following formula:

$$L_{\text{adv}}=\frac{E}{z-P_{\text{noise}}}[D(x)]-\frac{E}{z-P_{\text{noise}}}[D(G(z))]+\varphi_{\text{gp}}*\text{GP}.$$ (16)

Here, D(x) and D(G(z)) are the scores of the discriminator for the real data x and the generated data G(z), respectively. Traditional WGAN enforces Lipschitz continuity via weight clipping, but this can easily lead to gradient vanishing or explosion. The GP mechanism is applied to constrain the discriminator's gradient norm for the interpolated sample $\hat{x}$ to lie close to 1. The corresponding computation is performed according to the following formula:

$$\text{GP}=\frac{E}{\hat{x}-P_{\text{noise}}}[{\left({\left\|\nabla_{\hat{x}}D(\hat{x})\right\|}_{2}-1\right)}^{2}].$$ (17)

Here, $\hat{x}$ is the random linear interpolation of the real data and the production data [ $\hat{x}=\epsilon x+(1-\epsilon )G(z),\;\epsilon \cdot U(0,1)]$.

During model training, the generator and discriminator use an alternating optimization scheme. The discriminator is updated several times per round before the generator, gradually improving the authenticity and semantic consistency of the generated data. After training, C-GAN can generate fitting data that highly match the individual characteristics of patient samples whose clinical characteristics lack environmental exposure information.

### D. Dynamic gating mechanism

To address unbalanced modal contributions arising from individual differences in multimodal data fusion, a dynamic gating mechanism is applied.^32,33^ The learnable weight network is trained on the patient's clinical characteristics to adaptively adjust the fusion ratios of CT images, lung function indicators, and environmental exposure data.

The patient's clinical indicators are used as input, and the modal weight parameters are generated by the multilayer perceptron.^34,35^ The calculations are performed according to the following formula:

$$u=\text{MLP}(x)=W_{2}*\text{ReLU}(W_{1}*x+b_{1})+b_{2}.$$ (18)

Here, $W_{1}\in R^{h*2}$ (input layer to hidden layer); $W_{2}\in R^{3*h}$ (hidden layer to output layer); h is the number of hidden nodes.

The unnormalized weight $u\;[u_{\text{CT}},u_{\text{Lung}\;\text{function}},{\;W}_{\text{Environment}}]$ is normalized, as follows:

$$w_{i}=\frac{\text{exp}(u_{i})}{\sum_{j}\text{exp}(u_{j})},\;i\in \left(CT,\;\text{Lung}\;\text{function},\;\text{Environment}\right).$$ (19)

Using Formula (19), the dynamic weights $w_{CT}$, $w_{\text{Lung}\;\text{function}}$, and $w_{\text{Environment}}$ of each modal are obtained.

The normalized weights are applied to features $f_{CT}$, $f_{\text{Lung}\;\text{function}}$, and $f_{\text{Environment}}$ after cross-modal alignment. The feature fusion is calculated as follows:

$$f_{\text{Fusion}}=w_{CT}*f_{CT}+w_{\text{Lung}\;\text{function}}*f_{\text{Lung}\;\text{function}}+w_{\text{EnvironmentT}}*f_{\text{Environment}}.$$ (20)

The dynamic weight allocation embeds clinical prior knowledge into the fusion process through the Multilayer Perceptron-Sofmax gating network driven by patient attributes.

The dynamic gating mechanism and MMDF-Net adopt a joint training strategy. The cross-entropy loss for the main task, COPD risk stratification, is denoted by $L_{\text{cls}}$; the contrast loss for contrast learning cross-modal alignment is denoted by $L_{\text{contrast}}$; and the loss for C-GAN-generated environmental data is denoted by $L_{\text{WGAN}}$. The total loss is given by

$$L_{\text{total}}=L_{\text{cls}}+\lambda_{1}L_{\text{contrast}}+\lambda_{2}L_{\text{WGAN}}.$$ (21)

Here, $\lambda_{1}$ = 0.5 and $\lambda_{2}$= 0.2 are hyperparameters.

During training, the batch size is set to 32–64 to balance stability and efficiency. Dual-tower contrastive learning requires simultaneous processing of cross-modal samples, resulting in slightly higher sampling complexity than single-modal models, but stratified sampling can reduce redundant computations. Regarding data quality, CT images must have a slice thickness of 1 mm, a 512 × 512 pixel resolution, and no noticeable artifacts. Lung function indicators' (FEV1/FVC) detection error must be ≤5%, environmental exposure data missing rate ≤30%, and clinical attribute data (such as smoking history) label consistency ≥95%. Key feature information must be retained after preprocessing, such as histogram equalization and Z-score standardization.

Key hyperparameters include the contrastive learning temperature parameter T, the C-GAN gradient penalty coefficient φ_9p_, the number of hidden-layer nodes h in the dynamically gated MLP, the loss weights λ_1_ and λ_2_, and the ResNet-50 pre-trained weight fine-tuning strategy. The approach employed a combination of “domain initial screening + grid search + threefold cross-validation”: First, referencing experience from multimodal contrastive learning and WGAN-GP, T was limited to 0.01–0.1, φ_9p_ to 10, and h initially set to 64–256; then, a grid search was performed on λ_1_ (0.1–1.0), λ_2_ (0.1–0.5), and h, with validation set AUC and F1 as optimization targets, ultimately determining T = 0.05, h = 128, λ_1_ = 0.5, and λ_2_ = 0.2; the ResNet-50 pre-trained weights were selected to adapt to lung features in CT images by comparing the validation set performance of “freezing the feature extraction layer” and “fine-tuning the last three layers.”

## Data Availability

The data that support the findings of this study are available from the corresponding author upon reasonable request.
